# Does grandchild care intention, intergenerational support have an impact on the health of older adults in China? A quantitative study of CFPS data

**DOI:** 10.3389/fpubh.2023.1186798

**Published:** 2023-08-24

**Authors:** Xinjie Zhang, Wei Chen

**Affiliations:** ^1^Department of Public Administration, School of Management, Jiangsu University, Zhenjiang, China; ^2^Department of Social Medicine and Health Administration, School of Government, Nanjing University, Nanjing, China

**Keywords:** the “universal three-child” policy, grandchild care intention, intergenerational support, the health of older adults, multiple mediating effects

## Abstract

**Background:**

With the rising of fertility cost and the implementation of the “universal three-child” policy, the model of intergenerational support for grandchild is becoming an inevitable choice for more and more families in China. As the number of grandchildren increases and the interval between births extends, will the grandchild care intention and grandparents’ health be affected?

**Methods:**

Based on the data of China Family Panel Studies from 2018 and 2020, this study used multiple linear regression and multiple mediation tests to investigate the effect of grandchild care intention and intergenerational support on the health of older adults.

**Results:**

Firstly, actively taking care of grandchild has increased the self-rated health and mental health of older adults by 11.8 and 10.7%, respectively. Secondly, there is heterogeneity in the effect of intergenerational support from adults on health in their parents, among them, economic support improves the physical health by 5.5%; life care increases the self-rated and physical health by 3.3 and 0.8% respectively; emotional comfort improves the physical and mental health by 2.5 and 2.9%, respectively. Thirdly, grandchild care intention has a mediating effect on the health of older adults through economic support, life care, and emotional comfort.

**Conclusion:**

The grandchild care intention has positive effect on the health of older adults. The intergenerational supports (economic support, emotional comfort, and life care) have heterogeneous effects on the health of older adults, such as economic support mainly affects the physical health. Older adults who actively take care of their grandchild are more likely to gain intergenerational support and feedback from their adult children, transforming intergenerational support into a “win-win” model of resource reciprocity between generations. Based on this, it is necessary to re-establish the value identity of “caring for older adults” and “raising grandchildren” at the cultural level, continuously improve the fertility support policies at the government level and establish “caring for older adults” support platform at the social level.

## 1. Introduction

Grandchild care is an important issue of global concern, and China is no exception. In Chinese tradition, it is natural for grandparents to take care of their grandchild and children to support their parents (1). And older adults can prove their own value and alleviate the sense of loss brought by retirement in this process (2). However, due to factors such as economic development, social transformation, and changes in fertility attitudes, the relationship of intergenerational support is undergoing changes. Caring for grandchild seems to be the sole obligation and responsibility of older adults, which leads to a greater burden of caregiving and accompanying mental stress (3). In addition, more and more young people are leaving their homes at a young age and not return due to education, employment, and other reasons, the problem of “empty nest” is becoming increasingly severe, tragedies of older adults dying alone occasionally occur, taking care of grandchild seems to give them more reasons to gain support when they are old (4).

In July 2021, the “universal three-child” policy (which means that a couple can have three children) was officially implemented in China. It is anticipated that in the context of imperfect childcare services and fertility support policies, an increasing number of older adults will join the ranks of caring for grandchild, regardless of how many of them are voluntary, especially after experiencing the joys and sorrows of caring for their first grandchild (5). In addition, due to factors such as the delay of fertility time and the relaxation of fertility policy, the age gap between grandchildren continues to widen. Taking care of grandchild one after another not only takes up a large amount of social time and lowers the life quality of older adults, but also may cause serious damage to their health (6). So, in the complex context of deepening population aging, implementation of the “universal three-child” policy, and inadequate social services for older adults and children, how can we better play the role of intergenerational care under the premise of healthy aging? In view of this, based on the existing research, this study aims to analyze the association between grandchild care intention, intergenerational support and older adults’ health, so as to provide suggestions on how to achieve a positive interaction between “raising grandchildren” and “caring for older adults” from a theoretical perspective, and provide direction choices for achieving healthy aging.

## 2. Literature review

### 2.1. Grandchild care intention and the health of older adults

For a prolonged period, Chinese society has been characterized as an “immobile society” shaped by regional restrictions, with residents living within a certain range on a family basis, which greatly facilitates the intergenerational care. Grandparents often take care of their grandchild instead of their adult children partially or completely, which are called intergenerational care (7). However, the behavior of caring for their grandchild is not random, but rather a self-selected behavior, usually manifested as willingness whether to take care of their grandchild actively (8). Due to differences of culture and lifestyle between the East and West, East Asians and Latinos tend to view the caring for grandchild as a family responsibility and tradition, and their willingness is more stronger than Caucasians’ who place greater emphasis on personal life (9, 10). Li and Feng (5) conducted a study of 12 cities in China, which showed that 64% of grandparents expressed a willingness to continue taking care of the second grandchild (5). Older adults in rural areas, especially women, who are relative disadvantage of socio-economic status, often show a stronger desire to care for grandchild in exchange for future security (11).

According to the Role Enhancement Theory, when older adults with care preferences actively take on the responsibility of caring for their grandchild, they are more likely to find the sense of achievement and belonging from their grandchild’s growth, and perceive an increase in resources, role enrichment, and emotional satisfaction, thus playing a protective role in their health (12, 13). On the contrary, according to the Role Conflict Theory, older adults who passively care for their grandchild after repeated requests from their children are likely to experience role confusion and prone to conflict with their children, which may affect their physical, psychological and spiritual health (14, 15). Based on this, this study proposes Hypothesis 1:

*H1*: Actively taking care of grandchild has a significant positive impact on the health of older adults.

### 2.2. Intergenerational support and the health of older adults

Intergenerational care is a reciprocal exchange of time and emotional resources between generations, which plays a crucial role in the continuation of intergenerational relationship with family bonds as the core (16). For the father’s generation, intergenerational support mainly refers to the provision of assistance from their offspring, specifically in three aspects, namely economic support, life care, and emotional comfort (17). Although migration and mobility in modern society are changing family structure and lifestyle patterns, intergenerational support from adult children is still the main source of service for older adults (18, 19). Moreover, the exchange between “raising grandchildren” and “caring for older adults” seem to be constantly strengthening, that is grandparents establish good emotional relationship by actively caring for their grandchild, in order to increase current and future support when they are old (20). Generally speaking, the longer the duration of intergenerational care, the higher the expectations of intergenerational support from their adult children (21).

From the perspective of the path of intergenerational support affecting the health of older adults, it is mainly achieved by increasing net transfer payments from adult children and increasing social frequency (22). Older adults who care for grandchild are more likely to gain financial, life and emotional support from their adult children, which promotes physical and mental health (23, 24). However, it should be noted that taking care of grandchild can increase intergenerational interaction opportunities between adult children and older adults, but due to differences in educational concepts, lifestyles, and other aspects between the two generations, chronic conflicts may also arise, leading to the pressures of caregiving and causing health damage to older adults (25, 26). In addition, existing literature recognizes the positive impact of grandchild care intention on intergenerational support (20). Based on this, this study proposes Hypothesis 2:

*H2*: There is a mediating effect of intergenerational support on the impact of grandchild care intention on the health of older adults.

### 2.3. Research review

In summary, scholars have conducted some research on the relationship between grandchild care, intergenerational support and the health of older adults, which provides a beneficial reference for our study. However, in terms of specific content, few scholars have conducted in-depth analysis on the relationship between grandchild care intention, intergenerational support, and the health of older adults based on the motivation of “raising grandchildren.” In addition, the life expectancy of older adults has been extended under the background of aging population, and the implementation of the “universal three-child” policy and the inadequate service for older adults and children have made the discussion on the problem of “raising grandchildren” more important and complex. Compared with existing literature, the main contributions of this study are reflected in the following two aspects: On one hand, in the complex context of the deepening of population aging and the relaxation of fertility policy, this study takes the motivation of “raising grandchildren” as the starting point and examines the impact of the grandchild care intention on the self-rated, physical, and mental health of older adults. On the other hand, this study examines the mediating role of intergenerational support in the impact of grandchild care intention on the health of older adults, in order to achieve a positive interaction between “raising grandchildren” and “caring for older adults,” and to leverage the important value of the model of intergenerational care on the premise of healthy aging.

## 3. Research design

### 3.1. Data sources

The China Family Panel Studies (CFPS) is a significant nationwide multidisciplinary tracking survey project that was organized by the Institute of Social Science Survey, Peking University since 2010. This project encompasses 25 provinces, municipalities, and autonomous regions. The data used in this study were sourced from the fifth and sixth rounds of the CFPS. Its questions accurately fit the connotation of the dependent and independent variables, and meet the research needs of grandchild care intention, intergenerational support and the health of older adults. Based on the theme, the study selected older adults aged 60 and above in the database and obtained 13,132 valid samples. In addition, this study focuses on examining the impact of grandchild care intention on older adults’ health with grandchild care experiences. Therefore, based on the respondents’ answers to the question “Have you taken care of grandchild in the past 6 months?,” we further deleted the sample of older adults who did not take care of grandchild and excluded samples with missing values, the final sample size was 6,419.

### 3.2. Variables

#### 3.2.1. Dependent variable

The dependent variable of this study is the health of older adults. In order to comprehensively evaluate the health level of older adults, we drew upon the measurement dimensions of health in CFPS to evaluate the health level of older adults from both subjective and objective perspective (24, 27). Among them, subjective health mainly refers to the self-rated health of older adults, reflecting their subjective evaluation of health status, which is measured by the respondents’ answers to the question “How healthy do you think you are?” (1 = very unhealthy; 2 = less healthy; 3 = relatively healthy; 4 = very healthy). Objective health mainly includes physical and mental health. Physical health refers to the ability to perform activities of daily living (ADL), which was evaluated by whether the respondents can go outdoors, eat, perform kitchen activities, take transportation, go shopping, take a bath and do laundry independently, and we manipulated it into a three-categories’ variable (1 = completely not independent; 2 = partially independent; 3 = completely independent). Mental health is mainly measured by respondents’ answers to the question “How often did you feel depressed in the past week?” (1 = 5–7 days; 2 = 3–4 days; 3 = 1–2 days; 4 = less than 1 day). In order to facilitate the test of mediating effects, we used principal component analysis (PCA) to combine “self-rated health” “physical health” and “mental health” into a composite health factor. Upon incorporating the above variables into the principal component analysis, the value of KMO surpasses the threshold of 0.6, the value of p obtained from Bartlett’s sphericity test is less than 0.00, and the cumulative variance explained by the extracted factors surpasses 50%, indicating that the extracted factors are meaningfully related to the original items. Subsequently, the scores of the composite health factor is computed using Thomson regression method.

#### 3.2.2. Independent variable

The independent variable of this study is grandchild care intention. The provision of grandchild care among older adults is selective, the closer the older adults is to their adult children, the more likely they are to assist in caring for their grandchild (2, 28). Based on this, we define “having close relationship with adult children and caring for grandchild” as “actively caring for grandchild,” and “not having close relationship with adult children and caring for grandchild” as “passively caring for grandchild.” Since this study has deleted the sample of older adults who had no experience of caring for grandchild, it is only necessary to consider the relationship between older adults with their adult children. According to the respondents’ answers to the question “How close have you been to your adult children in the past 6 months?” (1 = very close; 2 = not very close; 3 = average; 4 = close; 5 = very close), those who responded with “close” “very close” are categorized as “actively caring for grandchild = 1″, while others are defined as “passively caring for grandchild = 0″.

#### 3.2.3. Mediating variable

This study aims to examine the mediating effect of intergenerational support between grandchild care intention and the health of older adults. We measure the intergenerational support based on the following questions: “In the past 6 months, how much assistance did your adult children provide you per month?” “In the past 6 months, how often did your children help you with housework or take care of your diet and life?” “In the past 6 months, how often did you communicate with your children by phone, text message, letter or email?,” which are operationalized as financial support, life care, and emotional comfort separately.

#### 3.2.4. Control variables

Referring to the existing literature, we control variables that have potential effects on the health of older adults. These variables are mainly identified at the individual and family levels. Individual-level variables include gender, age, household registration status, education, marital status, monthly income level, medical insurance. Family-level variables include the frequency of meeting with children and frequency of grandchild care.

### 3.3. Empirical model

We constructed the model of multiple linear regression to test the impact of grandchild care intention, intergenerational support on the health of older adults, the multiple regression equation is shown in Equation (1).


(1) $$H_{i}=\beta_{0}+\beta_{1}X_{i}+\beta_{2}M_{i}+\beta_{3}N_{i}+\theta_{i}$$

Here, $H_{i}$ represents the self-rated, physical and mental health of older adults. $X_{i}$ represents the grandchild care intention. $M_{i}$ is the mediating variable of intergenerational support, including financial support, life care and emotional comfort. $N_{i}$ represents the control variables, including gender, age, household registration status, education, marital status, monthly income level, medical insurance, the frequency of meeting with children and frequency of grandchild care. $\beta_{0}$ is a constant term. $\theta_{i}$ is the random disturbance term. $\beta_{1}$ and $\beta_{2}$ are the main regression parameters. The significance of $\beta_{1}$ stands for whether grandchild care intention has effects on the health of older adults, and the significance of $\beta_{2}$ stands for whether intergenerational support has effects on the health of older adults.

**Figure 1:**
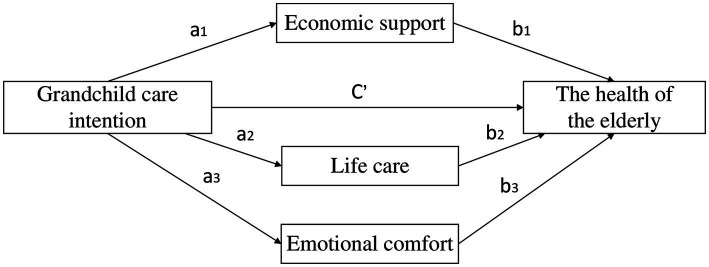
Multiple mediating effects model. a1 represents the effect of grandchild care intention on the economic support; a2 represents the effect of grandchild care intention on the life care; a3 represents the effect of grandchild care intention on the emotional comfort. b1 represents the effect of economic support on the health of the elderly under the control of grandchild care intention; b2 represents the effect of life care on the health of the elderly under the control of grandchild care intention; b3 represents the effect of emotional comfort on the health of the elderly under the control of grandchild care intention. c’ represents the effect of grandchild care intention on the health of the elderly under the control of intergenerational supports.

Subsequently, we used the Bootstrap method to examine the potential mediating effects of intergenerational support in the relationship between grandchild care intention and the health of older adults. This method can overcome the shortcomings of low statistical efficacy of the stepwise test and the Sobel test method, it can also effectively solve the problems of measurement errors of variables and the model of multiple mediating effects (29). The multiple mediating effects model constructed in this study is shown in Figure 1. The letters on the path of action indicate the regression coefficients between the variables.

As shown in Figure 1, $a_{i}$ represents the effect of grandchild care intention on each dimension of intergenerational support. $b_{i}$ denotes the effect of each dimension of intergenerational support on the health of older adults under the control of grandchild care intention variable. $c^{\prime }\;$ denotes the effect of grandchild care intention on the health of older adults under the control of intergenerational support. The mediating effect of grandchild care intention through financial support is expressed as $a_{1}*b_{1}$ . By analogy, the total mediating effect of the whole model is the sum of value of each mediating effect, denoted as $a_{1}*b_{1}+a_{2}*b_{2}+a_{3}*b_{3}$ .

## 4. Empirical results and analysis

### 4.1. Results

#### 4.1.1. Descriptive statistics

In order to provide an overview of dependent, independent and control variables, this study first conducted a descriptive statistical analysis, the results are shown in Table 1.

**Table 1:** Variable settings and descriptive statistics.

Variables	Definitions	Mean	S.D.	Type
Independent variable				
Grandchild care intention	Active care for grandchildren = 1, passive care for grandchildren = 0	0.77	0.419	V
Mediating variables				
Economic support	Amount of financial support: not provided = 1, less than $1,000 per month = 2, $1,000 and above per month = 3	1.84	0.720	D
Life care	Frequency of children helping with household chores or taking care of meals: never = 1, 1 day a few months = 2, 1 day a month = 3, 2–3 days a month = 4, 1–2 days a week = 5, 3–4 days a week = 6, almost every day = 7	3.46	2.397	D
Emotional comfort	Frequency of contact with children by phone, text messages: never = 1, 1 day a few months = 2, 1 day a month = 3, 2–3 days a month = 4, 1–2 days a week = 5, 3–4 days a week = 6, almost every day = 7	4.34	2.140	D
Dependent variables				
Self-rated health	You think your physical condition is: very unhealthy =1; less healthy =2; relatively healthy = 3; very healthy = 4	2.59	1.060	V
Physical health	The ability to perform daily activities such as exercise, eating, kitchen activities, transportation, shopping, cleaning, laundry: not independent at all = 1, partially independent = 2, fully independent = 3	2.63	0.650	D
Mental health	Frequency of feeling depressed in the past week: 5–7 days = 1; 3–4 days = 2; 1–2 days = 3; less than 1 day = 4	3.21	0.885	D
Control variables				
Age	60 ~ 74 years old = 0; age 75 and older = 1	0.09	0.291	V
Gender	Male = 1, Female = 0	0.48	0.500	V
Household Registration	Agricultural household = 1, non-agricultural household = 0	0.77	0.420	V
Education level	Elementary school and below =1, secondary school =2, college and above = 3	1.52	0.581	D
Marriage	Married = 1, not married = 0	0.85	0.360	V
Monthly income level	1,000 yuan and below =1, 1,001 ~ 3,999 yuan =2, 4,000 yuan and above =3	1.71	0.694	D
Medical Insurance	Participating in health insurance = 1, not participating in health insurance = 0	0.91	0.290	V
Frequency of meeting with children	Never =1, 1 day a few months =2, 1 day a month =3, 2–3 days a month =4, 1–2 days a week =5, 3–4 days a week =6, almost every day =7	6.14	1.356	D
Frequency of grandchild care	Never =1, 1 day a few months =2, 1 day a month =3, 2–3 days a month =4, 1–2 days a week =5, 3–4 days a week =6, almost every day =7	4.52	2.096	D
Sample size	6,419			

The variable type D represents virtual qualitative variables, and V represents virtual binary variables.

As shown in Table 1, the mean value of the grandchild care intention is 0.77, indicating that most grandparents are willing to take the initiative to care for their grandchild. The mean values of economic support, life care and emotional comfort are 1.84, 3.46, and 4.34, respectively, indicating that the majority of older adults have gained adequate intergenerational support from their adult children when they choose to care for their grandchild. In terms of the health of older adults, the mean values of self-rated, physical and mental health are 2.59, 2.63 and 3.21, respectively, indicating that most of older adults who provide grandchild care are in better health. Possibly because grandchild care is a less of drain on the older adults’ health; it is also possible that they are in good condition themselves and are thus able to provide grandchild care. In terms of the control variables, there are slightly more females (51.7%) than males (48.3%), a larger proportion of older adults with agricultural registration(77.1%) as opposed to non-agricultural registration(22.9%), the age distribution is 60 to 94 years old, with the majority in the age range of 60 to 74 years (90.7%), which indicates that female, agricultural and younger older adults are more likely to provide grandchild care. The results also show that the majority of older adults who provide grandchild care have married (84.7%), an education level of high school or less (95.6%), and a monthly income of less than $4,000 (86.2%). The frequency of grandchild care and the frequency of meeting with children are relatively high for older adults, with mean values of 4.52 and 6.14, respectively.

#### 4.1.2. Multiple regression

To analyse the impact of grandchild care intention, intergenerational support on the health of older adults, this study conducted multiple linear regression analysis. The multiple regression results in Table 2 show that grandchild care intention has a significant positive effect on both self-rated and mental health of older adults, actively caring for grandchild significantly increased the self-rated and mental health of older adults by 11.8% and 10.7%, respectively, at a statistical level of 0.1%. Therefore, Hypothesis 1 is validated. There is heterogeneity in the impact of intergenerational support on the health of older adults. Specifically, the economic support has significant positive effect on the physical health, manifested as an increase of 5.5% for each level of economic support, while the impact of economic support on self-rated and mental health is not significant. The life care has significant positive effect on the self-rated and physical health, and with each increase in the frequency of life care, the self-rated and physical health of older adults increase by about 3.3% and 0.8%, respectively. However, the impact of life care on mental health is not significant. The emotional comfort has a significant positive impact on the physical and mental health, manifested as an increase of about 2.5% and 2.9% for each level of emotional comfort, while the impact of emotional comfort on self-rated health is not significant.

**Table 2:** The multiple regression results.

Variables	Self-rated health	Physical health	Mental health
Grandchild care intention	0.118***	0.037	0.107***
Economic support	0.028	0.055***	0.031
Life care	0.033***	0.008*	0.001
Emotional comfort	0.007	0.025***	0.029***
Age	−0.013	−0.155***	0.062
Gender	0.148***	0.085***	0.157***
Household registration	0.187***	−0.052**	−0.082**
Education level	0.152***	0.033*	0.004
Marriage	0.002	0.006	0.036
Monthly income level	0.618***	0.112***	0.184***
Medical insurance	−0.131**	0.368***	0.269***
Frequency of grandchild care	−0.039***	0.009	0.007
Frequency of meeting with children	0.008	−0.006	0.018**
Constant	1.127***	1.927***	2.143***
Sample size	6,419	6,419	6,419

*p < 0.05, **p < 0.01, ***p < 0.001.

From the relationship between the control variables and the health of older adults, the regression results show that men and high pensioners have higher level of self-rated, physical and mental health. Older adults with agricultural registration have better self-rated health, but their physical and mental health is worse. Participating in medical insurance lowers the self-rated health of older adults, but improves their physical and mental health. Compared to older adults under 75 years old, the physical health of older adults over 75 years old has improved by about 15.5%. For each level of education improvement, the self-rated and physical health of older adults increase by about 15.2% and 3.3%, respectively. For each level of increase in the frequency of caring for grandchild, the older adults’ self-rated health decreases by approximately 3.9%. For each level of increase in frequency of meeting with children, the mental health of older adults increases by approximately 1.8%.

#### 4.1.3. Mediating effects test

To explore the mechanism underlying the impact of grandchild care intention on the health of older adults, we used the SPSS macro program compiled by Hayes (30) to test the model of multiple mediating effects, and then used the non-parametric percentage Bootstrap method with deviation correction to examine the significance level of mediating effects. As shown in Table 3, the grandchild care intention has positive effect on economic support, life care and emotional comfort. When the grandchild care intention and economic support, life care, emotional comfort jointly predict the health of older adults, the economic support, life care and emotional comfort still have significant positive effect on the health of older adults. The model of multiple mediating effects passed the test.

**Table 3:** Regression analysis of multiple mediation.

Regression equation		Overall fit index		Regression coefficient	
Result variable	Forecast variables	*R*	*R* ^2^	*F*	*β*
Economic support	Grandchild care intention	0.133	0.017	115.624	0.229***
Life care	Grandchild care intention	0.112	0.013	81.167	0.640***
Emotional comfort	Grandchild care intention	0.205	0.042	282.346	1.050***
Health	Grandchild care intention	0.194	0.038	62.473	0.037***
	Economic support				0.013**
	Life care				0.005***
	Emotional comfort				0.011***

*p < 0.05, **p < 0.01, ***p < 0.001.

**Figure 2:**
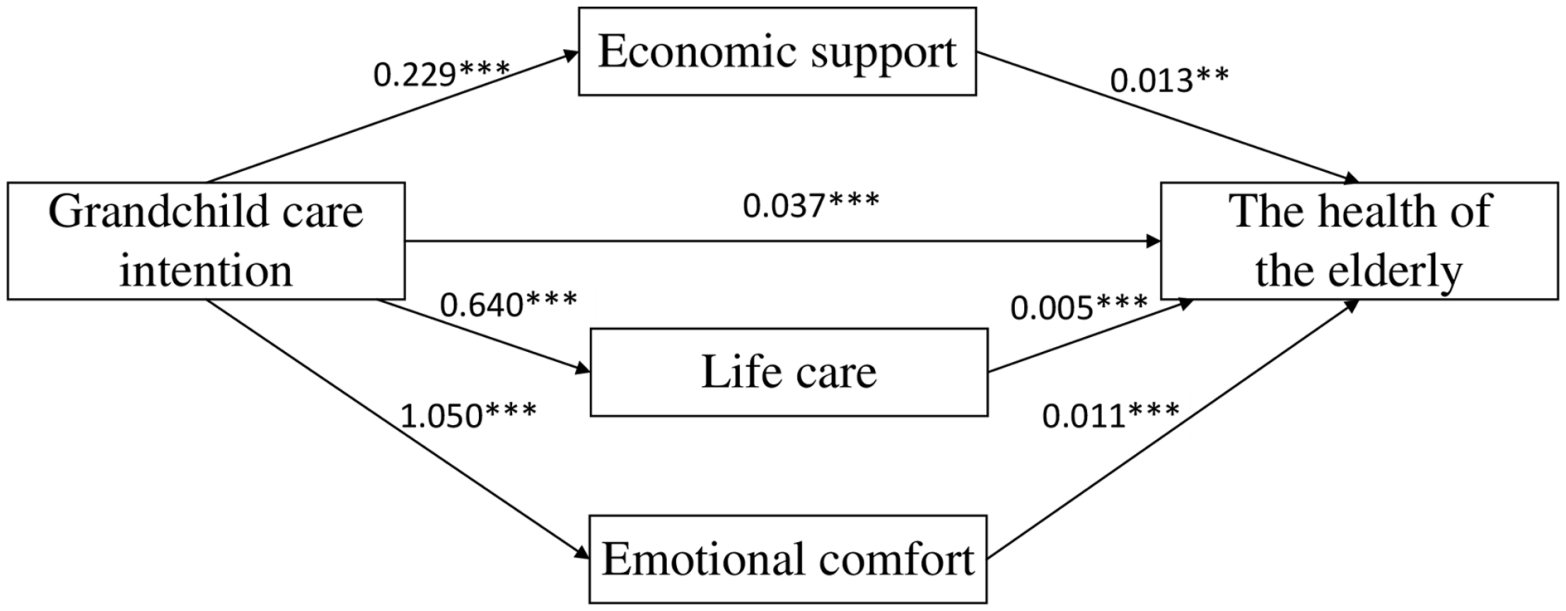
Diagram of chain mediation (*p < 0.05, **p < 0.01, ***p < 0.001).

We further used the Bootstrap method proposed by Fang et al. to test the mediating effects (31). This method requires sampling of 5,000 times and then calculates the 95% confidence interval. If the confidence interval does not include 0, it means that the mediating effect is significant. The results in Table 4 show that the mediating effects of economic support, life care and emotional comfort are significant, and the value of total mediating effects is 0.0177, that is $a_{1}*b_{1}+a_{2}*b_{2}+a_{3}*b_{3}=0.0177$ . Specifically, the mediating effects are generated through three chains: the first indirect effect is manifested as grandchild care intention → economic support → health of older adults (0.0029); the second indirect effect is manifested as grandchild care intention →life care → health of older adults (0.0029); the third indirect effect is manifested as grandchild care intention →emotional comfort → health of older adults (0.0119). The 95% Bootstrap confidence interval of the above intermediary paths does not contain 0, which indicates that the mediating effects of economic support, life care, and emotional comfort are statistically significant, and thus Hypothesis 2 is verified. The detailed path coefficient is shown in Figure 2.

**Table 4:** Results of mediating effects.

	Effect value	Boot S.E.	Boot CI lower limit	Boot CI upper limit	Relative mediating effect
Total indirect effect	0.0177	0.0017	0.0145	0.0210	32.30%
Indirect effect 1	0.0029	0.0008	0.0014	0.0046	5.29%
Indirect effect 2	0.0029	0.0007	0.0015	0.0044	5.29%
Indirect effect 3	0.0119	0.0014	0.0092	0.0148	21.72%

### 4.2. Discussion and conclusion

Currently, China is confronted with multiple challenges such as rapid population changes, reforms in fertility policies, and the invasion of the “silver wave,” intergenerational care is not only an inevitable choice for families to cope with the pressures of childbearing, but also an important expression of social participation among older adults. Based on the data of China Family Panel Studies (CFPS) from 2018 and 2020, this study used the model of multiple linear regression and multiple mediating effects to explore the impact of grandchild care intention and intergenerational support on the health of older adults and its mechanism. Some interesting discussion and the main conclusion are as follows.

Firstly, the grandchild care intention has significant positive impact on the self-rated and mental health of older adults. Older adults who actively take care of their grandchild usually suffer less psychological stress, and can obtain satisfaction and pleasure in the process of raising grandchildren. While the impact of grandchild care intention on the physical health is not significant. A plausible explanation is that the physical health of older adults is rigid to some extent and less susceptible to short-term emotional shifts, but more affected by long-term and ongoing environments of family, social, and work.

Secondly, there is heterogeneity in the impact of intergenerational support provided by adult children on the health of older adults. The economic support has significant positive impact on the physical health of older adults, while the impact on self-rated and mental health is not significant. It may be related to the fact that economic support can just meet the daily medical and healthcare needs of older adults, while self-rated and mental health are more in need of the companionship and care provided by adult children. The life care positively affects the self-rated and physical health of older adults, while the effect on mental health is not significant. The reason is that although life care can improve the overall life quality of older adults to some extent, it also weakens the self-esteem and autonomy of them. In terms of emotional comfort, it has positive impact on the physical and mental health of older adults, while the effect on self-rated health is not significant. A possible explanation is that the measurement of emotional comfort is primarily based on the frequency of communicate between older adults with their children in the CFPS questionnaire, and may have not included all factors of children’s concern for their parents.

Thirdly, Under the control of gender, age, household registration, education, monthly income, medical insurance, the frequency of grandchild care and the frequency of meeting with children, this study finds that economic support, life care, and emotional comfort all play a mediating role in the relationship between grandchild care intention and the health of older adults. Older adults who take the initiative to care for their grandchild are more likely to gain intergenerational support and feedback from their adult children, thus forming a resource reciprocity relationship between offspring and parents, which not only benefits the family’s function of “caring for older adults” and “raising grandchildren,” but also has positive impact on the health of older adults.

## 5. Suggestions

To optimize the positive impact of intergenerational care on the health of older adults, we propose several suggestions. From a cultural perspective, re-establish the value identity of “caring for older adults” and “raising grandchildren.” On one hand, encourage older adults to actively care for their grandchild. On the other hand, guide adult children to actively provide economic support, life care, and emotional comfort to their parents, so that both children and grandparents can enhance their relationship of family interaction by providing two-way support within their abilities. At the government level, we suggest continuously improving the fertility support policies, such as actively carrying out inclusive construction for childcare, reducing the pressures of “caring for children” on women at childbearing age, so that they can put more time, material and other resources into “caring for older adults.” At the social level, establish “caring for older adults” support platform to adapt to the constantly evolving socio-economic environment. For example, continuously strengthen social support for older adults, encourage social organizations, enterprises and volunteers to provide services necessary for older adults in their daily life. Another suggestion is to provide flexible support for older adults with the community as the carrier. Community workers and volunteers provide special assistance for older adults, especially those who care for grandchild, such as physical examination, emotional communication, assistance in handling necessary things and so on, so as to alleviate the mental and emotional pressures on older adults, to truly form a positive interaction between “caring for older adults” and “raising grandchildren,” and to promote the realization of the goal of healthy aging and the relaxation of fertility policy.

## Data availability statement

The datasets presented in this study can be found in online repositories. The names of the repository/repositories and accession number(s) can be found in the article/supplementary material.

## Author contributions

XZ was responsible for the research design, completed the model design, and wrote the initial manuscript. WC was responsible for the literature search, data analysis, original draft writing, and supervision. All authors have read and agreed to the published version of the manuscript.

## Funding

The research was supported by the National Natural Science Foundation of China (Grant No. 71804061), the National Natural Science Foundation of China (Grant No. 72174078), and the ‘Qing Lan Project’ of Jiangsu Universities (2022).

## Conflict of interest

The authors declare that the research was conducted in the absence of any commercial or financial relationships that could be construed as a potential conflict of interest.

## Publisher’s note

All claims expressed in this article are solely those of the authors and do not necessarily represent those of their affiliated organizations, or those of the publisher, the editors and the reviewers. Any product that may be evaluated in this article, or claim that may be made by its manufacturer, is not guaranteed or endorsed by the publisher.
